# Prevalence and bedside predictors of difficult direct laryngoscopy among 3,080 adult elective surgical patients at a Cambodian tertiary center: a retrospective cohort study

**DOI:** 10.1186/s12871-026-03846-4

**Published:** 2026-04-24

**Authors:** Nasin Pa, Leabheng Bunly, Vibopha Srey, Saly Saint, Makara Chin, Sarath Phoeun, Moni Rath Heng, Sovannara Leng

**Affiliations:** 1 Department of Anesthesiology and Critical Care, Preah Ang Duong Hospital, Phnom Penh, Cambodia; 2 Faculty of Medicine, University of Health Sciences, Phnom Penh, Cambodia

**Keywords:** Airway Management, Laryngoscopy, Intubation, Intratracheal, Mallampati test, Body Mass Index, Predictive Value of Tests, Cambodia

## Abstract

**Background:**

Failure to anticipate difficult direct laryngoscopy (DDL) leads to catastrophic airway events. While prediction tools have been developed primarily in European and North American cohorts, evidence from Southeast Asia—particularly in settings with high volumes of head-and-neck pathology—is limited. We investigated DDL prevalence, identified independent bedside predictors, and evaluated a simplified cumulative risk score in a Cambodian tertiary center.

**Methods:**

We conducted a retrospective cohort study of 3,080 consecutive adults undergoing elective surgery with planned Macintosh direct laryngoscopy (January–June 2023) at Preah Ang Duong Hospital, Phnom Penh. DDL was defined as Cormack–Lehane grade III/IV or three or more laryngoscopic attempts. Seven bedside predictors were analyzed using multivariable logistic regression. A simplified composite risk score (range 0–3) was derived from the strongest independent predictors and evaluated using receiver operating characteristic (ROC) analysis. To assess confounding by surgical case-mix, an additional model adjusted for head-and-neck surgical category. Model calibration was assessed with the Hosmer–Lemeshow goodness-of-fit test. Data were analyzed using SPSS V30.

**Results:**

DDL prevalence was 9.03% (278/3,080; 95% CI: 8.01–10.04%), rising to 13.53% in maxillofacial and 11.34% in ENT procedures. The mean BMI was 26.5 ± 6.4 kg/m², with 35.0% classified as obese using the WHO Asian-specific threshold (≥ 27.5 kg/m²). In multivariable analysis, six independent predictors emerged: Mallampati III/IV (adjusted odds ratio [AOR] 4.15; 95% CI: 3.02–5.70), BMI ≥ 27.5 kg/m² (AOR 2.92; 95% CI: 2.25–3.79), limited neck mobility (AOR 2.13; 95% CI: 1.64–2.75), thyromental distance ≤ 6.5 cm (AOR 1.95; 95% CI: 1.46–2.59), neck circumference ≥ 40 cm (AOR 1.41; 95% CI: 1.09–1.84), and inter-incisor gap ≤ 3 cm (AOR 1.41; 95% CI: 1.06–1.88); all *p* < 0.02. The upper lip bite test was not independently predictive (*p* = 0.91). All six predictors retained significance after adjustment for head-and-neck surgical category (H&N AOR 2.42; 95% CI: 1.83–3.21). A simplified composite score (Mallampati + BMI + TMD) achieved an AUC of 0.72 (95% CI: 0.69–0.75; bootstrap-corrected 0.72), with 82.7% sensitivity, 54.0% specificity, and 96.9% negative predictive value at a cutoff of 2 or greater. The score demonstrated a clear dose–response relationship (DDL prevalence: 2.2% at score 0, 3.3% at score 1, 11.9% at score 2, 24.7% at score 3). The model showed acceptable calibration (Hosmer–Lemeshow *p* = 0.42). Pre-specified sensitivity analyses confirmed score stability in non-head-and-neck cases (AUC 0.75; *n* = 1,488) and under a Cormack–Lehane III/IV–only definition (AUC 0.72). Specialty-specific AUCs ranged from 0.68 to 0.82, supporting generalizability across surgical subgroups.

**Conclusions:**

DDL affects nearly 1 in 11 elective surgical patients in this high-acuity cohort, driven largely by the case-mix of head-and-neck procedures. A simple, three-component bedside score offers a zero-cost tool to enhance preoperative risk stratification in resource-limited settings, with BMI emerging as a particularly strong predictor when assessed using population-specific thresholds. Prospective, multicenter validation is required before routine clinical implementation.

**Supplementary Information:**

The online version contains supplementary material available at 10.1186/s12871-026-03846-4.

## Introduction

Securing the airway is the most time-critical step in anesthetic management. Failure can precipitate —within minutes— hypoxemia, aspiration, brain injury, or death, making difficult direct laryngoscopy (DDL)—defined here as Cormack–Lehane grade III/IV or the need for three or more laryngoscopic attempts—a major patient-safety concern [1–3]. Unexpected difficulties during endotracheal intubation remain a primary concern in general anesthesia and can lead to catastrophic outcomes [4–7].

The prevalence of DDL varies widely (1.5–18%) across studies and settings [8]. For example, the DIFFICAIR trial reported a 1.86% prevalence of unanticipated difficult intubation in a European multicenter cohort, whereas studies from Ethiopia and India reported 12.2% and 2.6–2.9%, respectively, underscoring the influence of patient demographics, surgical case-mix, and practice patterns [8–10]. Since no single predictor reliably identifies all DDL cases, combining anatomical, physiological, and demographic factors is essential to improve prediction accuracy [11, 12]. In some cohorts, use of the Laryngoscopic Exam Test (LET) demonstrated a prevalence of 6.1%, further highlighting heterogeneity and the need for context-specific tools [13–15].

Preoperative airway assessment allows tailored strategies and equipment selection. Common bedside tools include the Mallampati classification, thyromental distance (TMD), neck mobility, Upper Lip Bite Test (ULBT), and neck circumference (NC). However, their sensitivity is often limited; while Mallampati and ULBT can show high specificity (up to 92%), inadequate sensitivity contributes to undetected cases [11–16]. Notably, one large study of 188,064 patients registered in the Danish Anaesthesia Database reported that 93% of difficult intubations were not predicted by routine preoperative assessment [5, 17]. Multivariate and ratio-based models—such as the neck-circumference-to-thyromental-distance (NC/TMD) ratio—have shown promise for improving prediction, including in obese and non-obese populations [11, 18–20].

Compared with the extensive airway prediction literature from Europe, North America, and parts of South Asia, research from Southeast Asia remains limited. To date, no study has evaluated combined bedside predictors in a Cambodian cohort, where the population’s craniofacial morphology, body habitus, and disease profiles may differ meaningfully from those in other geographic regions. This gap is particularly important given that resource-limited hospitals may lack video laryngoscopes and fiberoptic equipment, making accurate preoperative identification of at-risk patients essential for safe planning [11, 18, 21].

This study was conducted at Preah Ang Duong Hospital, a national referral center with a high volume of otolaryngology (ENT) and maxillofacial procedures—settings inherently associated with increased airway difficulty due to anatomical distortion from tumors, goiters, and facial trauma [22–24]. Within the eligible study cohort (January–June 2023), ENT (*n* = 794; 25.78%) and maxillofacial (*n* = 798; 25.90%) surgeries comprised over half (51.7%) of procedures, a distinguishing feature of this population that was likely to elevate baseline DDL rates compared with general surgical populations.

### Objectives

Accordingly, this retrospective cohort study aims to: (i) quantify the prevalence of DDL in adult elective surgical patients at this tertiary center; (ii) identify independent bedside predictors of DDL—including BMI, Mallampati class, TMD, ULBT, neck mobility, NC, and inter-incisor gap (IIG)—using multivariable logistic regression; and (iii) determine whether a simplified composite score derived from the strongest predictors improves discrimination beyond individual tests, to refine preoperative risk stratification and inform institutional airway algorithms in resource-constrained Southeast Asian settings.

## Methods

### Study design and setting

We conducted a retrospective cohort study analyzing data from 1 January to 30 June 2023 at Preah Ang Duong Hospital, Phnom Penh, Cambodia. The hospital serves as a national referral center for otorhinolaryngology and maxillofacial surgery. The study protocol was approved by the National Ethics Committee for Health Research (NECHR), Cambodia (Reference Number: 478; Approval Date: 11 December 2024) and the Preah Ang Duong Hospital Research Ethics Committee. The institutional ethics committee waived the requirement for informed consent due to the retrospective analysis of de-identified routine clinical data. This study is reported in accordance with the Strengthening the Reporting of Observational Studies in Epidemiology (STROBE) guidelines for cohort studies [25]; a completed checklist is provided as Supplementary Table S1.

### Participants

The cohort included consecutive adult patients (18 years or older) scheduled for elective surgery under general anesthesia requiring tracheal intubation via planned Macintosh direct laryngoscopy.

### Exclusion criteria

**Fig. 1:**
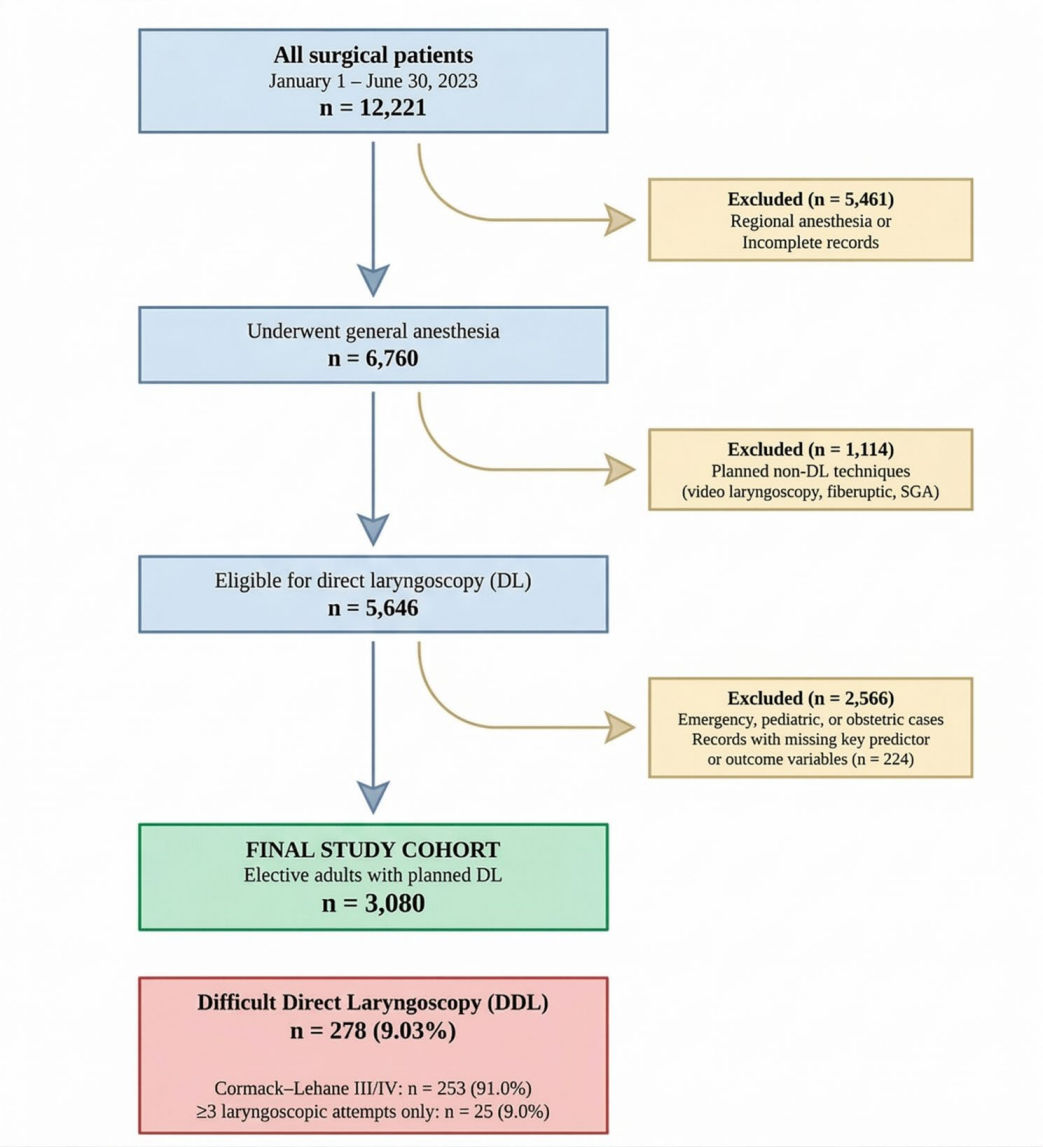
Study flowchart (patient selection). Patient selection from 12,221 total surgical patients down to 3,080 final cohort, with exclusion reasons at each step and the DDL breakdown (278 total: 253 by C-L III/IV, 25 by ≥ 3 attempts only)

Of 5,646 eligible patients scheduled for direct laryngoscopy, 2,342 were excluded due to ineligibility (pediatric, emergency, or obstetric cases). A further 224 patients (7.3% of the remaining eligible population) were excluded due to incomplete documentation of key airway predictors or outcome variables. The final analysis included 3,080 adult elective patients (Fig. 1). To assess potential selection bias, we compared available demographic characteristics (age, sex) between included and excluded patients; no statistically significant differences were found.

### Data collection and predictor definitions

Preoperative airway assessments were extracted from a standardized departmental anesthesia record that included dedicated fields for each airway predictor. This structured form served as the institutional protocol for systematic airway evaluation and was completed for all patients undergoing general anesthesia. All assessments were performed by attending anesthesiologists or supervised trainees as part of routine preoperative evaluation. Trainees received instruction in airway assessment techniques during their orientation and were directly supervised by attending anesthesiologists; however, formal inter-rater reliability calibration exercises were not conducted, which is acknowledged as a limitation.

Additional demographic and clinical variables recorded included age, sex, body mass index, American Society of Anesthesiologists (ASA) physical status classification, surgical specialty, and primary surgical indication. Surgical indications were categorized as malignancy, trauma or fracture, benign structural lesion (e.g., goiter, benign tumor, congenital anomaly), or other, based on the operative diagnosis recorded in the anesthesia chart.

We analyzed seven bedside predictors using a priori high-risk cutoffs. Mallampati class was assessed with the patient seated upright, mouth maximally opened, and tongue protruded without phonation, and graded I through IV using the modified Samsoon–Young classification [26]; classes III and IV were defined as high risk. Body mass index (BMI) was calculated from height and weight recorded at the preoperative visit; a cutoff of 27.5 kg/m² or greater was applied in accordance with the WHO Asian-specific threshold for obesity. Thyromental distance (TMD) was measured with a rigid ruler from the thyroid notch to the bony point of the mentum with the head fully extended and the mouth closed; a value of 6.5 cm or less was defined as high risk. Inter-incisor gap (IIG) was measured as the maximal distance between the upper and lower incisor edges during voluntary mouth opening, using a ruler or caliper; a value of 3 cm or less was defined as high risk. Neck mobility was assessed by clinical estimation of maximal head extension from the neutral position by the assessing anesthesiologist and classified as restricted (less than 80 degrees) or normal; no goniometer was used, and this visual estimation method may introduce inter-observer variability. Neck circumference (NC) was measured at the level of the thyroid cartilage with a flexible measuring tape while the patient sat with the head in the neutral position; a value of 40 cm or greater was defined as high risk. The Upper Lip Bite Test (ULBT) was graded I through III according to the patient’s ability to cover the upper lip vermilion with the lower incisors; Grade III (complete inability to bite the upper lip) was classified as high risk.

### Outcome measures

The primary outcome was difficult direct laryngoscopy (DDL), defined as either: (1) a modified Cormack–Lehane (C-L) glottic view of Grade III or IV, or (2) the requirement for three or more laryngoscopic attempts. All laryngoscopies were performed or directly supervised by attending anesthesiologists. In cases involving trainees, the attending confirmed the Cormack–Lehane grade and, where applicable, completed the intubation. The attempt count comprised all laryngoscopic insertions regardless of whether initiated by a trainee or attending; however, each attempt was performed under direct attending supervision, and the final grading decision rested with the attending anesthesiologist. This inclusive definition was chosen to reflect real-world clinical practice at our institution [27, 28].

### Statistical analysis

Data were analyzed using IBM SPSS Statistics version 30 (IBM Corp., Armonk, NY). Continuous variables were summarized as mean ± standard deviation (SD) and categorical variables as frequencies and percentages.

Binary logistic regression was selected as the primary analytic method because the outcome (DDL) is dichotomous, the predictors were pre-specified and dichotomized at clinically established cutoffs, and logistic regression remains the standard approach in the airway prediction literature — facilitating direct comparison with prior composite scores such as SARI, LEMON, and the Wilson risk sum [5, 8, 29–31]. Alternative machine-learning approaches (e.g., LASSO penalized regression, random forest, gradient-boosted trees) may improve discrimination or variable selection but require larger development and validation samples to avoid overfitting, and their outputs are less interpretable at the bedside. These methods should be explored in future prospective studies with adequate sample sizes for split-sample or external validation.

#### Model development

Univariate associations between each predictor and DDL were tested using Chi-square or Fisher’s exact tests. Variables with *p* < 0.05 on univariate analysis were entered into a multivariable logistic regression model to identify independent predictors. Multicollinearity was assessed using variance inflation factors (VIF), with a threshold of VIF less than 5 considered acceptable. Adjusted odds ratios (AOR) with 95% confidence intervals were reported. To evaluate potential confounding by surgical case-mix, we performed an additional multivariable model that included head-and-neck surgical category (ENT or maxillofacial versus all other specialties) as a binary covariate alongside the seven bedside predictors. This allowed us to determine whether the identified predictors retained independent significance after accounting for the inherently higher DDL risk associated with head-and-neck procedures. Granular pathological diagnosis (e.g., specific cancer type, fracture classification) was not routinely coded in a standardized manner suitable for multivariable modeling and could not be included as a covariate; this is acknowledged as a limitation.

#### Composite score construction

The composite score (range 0–3) was derived by assigning one point for each of the three strongest independent predictors that are most feasible for bedside application: Mallampati class III/IV, BMI 27.5 kg/m² or greater, and TMD 6.5 cm or less. These three were selected because they were among the strongest predictors in multivariable analysis (AOR range 1.95–4.15, all *p* < 0.001), they represent distinct anatomical domains (oropharyngeal visibility, body habitus, and mandibular space) thereby reducing redundancy, and they are measurable with minimal equipment, making the score feasible in resource-limited settings. Although neck mobility (AOR 2.13) was also strongly associated with DDL, it was excluded from the simplified score because its measurement relies on clinical visual estimation that may introduce greater inter-rater variability, potentially reducing reliability when used by non-specialist providers. An equal-weighting approach (one point per predictor) was chosen for simplicity and clinical usability, acknowledging that a weighted score based on regression coefficients could improve discrimination modestly and should be explored in validation studies. Receiver operating characteristic (ROC) analysis determined the optimal decision threshold. A score of 2 or greater was defined as “high risk,” balancing sensitivity and specificity for screening purposes in this resource-limited context.

#### Model performance

Discrimination was assessed using the area under the receiver operating characteristic curve (AUC) with 95% confidence intervals. The performance of the composite score was compared against individual predictors. Calibration was evaluated using the Hosmer–Lemeshow goodness-of-fit test. To assess potential over-optimism in the AUC estimate, we performed 1,000-iteration bootstrap resampling and report the bias-corrected AUC. A two-sided p-value < 0.05 was considered statistically significant.

#### Sample size considerations

With 278 DDL events among 3,080 patients, the study provides approximately 40 events per candidate variable (278/7), exceeding the commonly cited minimum of 10–20 events per variable for stable logistic regression estimates [32].

#### Sensitivity analyses

To assess the robustness of findings, we performed three pre-specified sensitivity analyses: (1) restricting the cohort to non-head-and-neck surgical cases (excluding ENT and maxillofacial procedures) to evaluate whether the predictors and composite score remain valid in a general surgical population; (2) defining DDL solely by Cormack–Lehane grade III/IV (excluding cases classified as DDL based only on three or more attempts) to test consistency across outcome definitions; and (3) evaluating the composite score’s discrimination (AUC with 95% confidence intervals) within individual surgical specialty subgroups where the number of DDL events permitted stable estimation (minimum 20 events), to serve as an internal validation of the score’s generalizability across case-mix categories.

## Results

### Demographic and surgical characteristics

3,080 consecutive adult patients underwent elective surgery with planned direct laryngoscopy during the study period (Table 1). The mean age was 53.2 ± 20.7 years, with a balanced sex distribution (49.3% male). The mean body mass index was 26.5 ± 6.4 kg/m²; applying the Asian-specific cutoff (≥ 27.5 kg/m²), 1,077 patients (35.0%) were classified as obese. The majority of patients were ASA physical status II (38.0%) or III (36.0%), with 12.0% classified as ASA IV. The case-mix was predominantly head-and-neck focused, with maxillofacial (25.9%) and ENT (25.8%) surgeries accounting for over half (51.7%) of the procedures. Laryngoscopies were performed by 12 attending anesthesiologists (mean experience: 8.5 ± 4.2 years post-residency) and 15 supervised anesthesiology trainees (PGY 1–4). All trainees were directly supervised by an attending anesthesiologist who confirmed the Cormack–Lehane grades.

**Table 1:** Baseline characteristics of the study cohort, stratified by DDL status

Characteristic	Overall (*N* = 3,080)	DDL (*n* = 278)	Non-DDL (*n* = 2,802)	*P*-value
Age (years), mean ± SD	53.2 ± 20.7	52.1 ± 20.2	53.3 ± 20.7	0.350
Male sex, n (%)	1,519 (49.3)	142 (51.1)	1,377 (49.1)	0.580
BMI (kg/m²), mean ± SD	26.5 ± 6.4	32.4 ± 9.6	25.9 ± 5.7	< 0.001
BMI ≥ 27.5 kg/m², n (%)	1,077 (35.0)	163 (58.6)	914 (32.6)	< 0.001
ASA physical status, n (%)				< 0.001
ASA I	432 (14.0)	21 (7.6)	411 (14.7)	
ASA II	1,170 (38.0)	86 (30.9)	1,084 (38.7)	
ASA III	1,108 (36.0)	84 (30.2)	1,024 (36.5)	
ASA IV	370 (12.0)	87 (31.3)	283 (10.1)	
Head-and-neck surgery, n (%)	1,592 (51.7)	198 (71.2)	1,394 (49.8)	< 0.001
Mallampati III/IV, n (%)	1,698 (55.1)	227 (81.7)	1,471 (52.5)	< 0.001
TMD ≤ 6.5 cm, n (%)	1,848 (60.0)	204 (73.4)	1,644 (58.7)	< 0.001
Neck mobility < 80°, n (%)	1,229 (39.9)	158 (56.8)	1,071 (38.2)	< 0.001
NC ≥ 40 cm, n (%)	1,090 (35.4)	118 (42.4)	972 (34.7)	0.012
IIG ≤ 3 cm, n (%)	2,030 (65.9)	200 (71.9)	1,830 (65.3)	0.031
ULBT Grade III, n (%)	1,445 (46.9)	141 (50.7)	1,304 (46.5)	0.204

Continuous variables compared using independent-samples t-test; categorical variables using Chi-square test

*DDL *Difficult direct laryngoscopy, *SD* Standard deviation, *BMI *Body mass index, *ASA *American Society of Anesthesiologists, *TMD *Thyromental distance, *NC *Neck circumference, *IIG *Inter-incisor gap, *ULBT *Upper Lip Bite Test

### Prevalence of difficult direct laryngoscopy

The overall prevalence of DDL was 9.03% (*n* = 278; 95% CI: 8.01–10.04%). Of the 278 DDL cases, 253 (91.0%) met the Cormack–Lehane III/IV criterion and 25 (9.0%) were classified as DDL solely based on three or more laryngoscopic attempts despite a Cormack–Lehane grade I or II view. Analysis by surgical specialty revealed significant heterogeneity (Table 2). DDL rates were highest in maxillofacial surgery (13.53%; 95% CI: 11.33–16.08%) and ENT (11.34%; 95% CI: 9.31–13.73%), compared with gynecology (3.17%; 95% CI: 1.68–5.91%). Using gynecology as the reference category, maxillofacial surgery carried the highest odds of DDL (OR 4.78; 95% CI: 2.39–9.58; *p* < 0.001), followed by ENT (OR 3.91; 95% CI: 1.94–7.86; *p* < 0.001). These findings confirm that the elevated overall DDL prevalence is driven predominantly by the high proportion of head-and-neck procedures, which together comprised 51.7% of all cases.

**Table 2:** DDL prevalence by surgical specialty with odds ratios (reference: Gynecology)

**Panel A: DDL prevalence by surgical specialty**					
**Surgical Specialty**	**Total Cases, n**	**DDL Cases, n**	**Prevalence, %**	**95% CI, %**	**Proportion of Cohort, %**
Maxillofacial	798	108	13.53	11.33–16.08	25.90
ENT	794	90	11.34	9.31–13.73	25.78
Neurosurgery	151	11	7.28	4.12–12.57	4.90
Abdominal	449	31	6.90	4.91–9.63	14.57
Urology	315	16	5.08	3.15–8.09	10.23
Orthopedic	289	13	4.50	2.65–7.54	9.38
Gynecology	284	9	3.17	1.68–5.91	9.22
Overall	3,080	278	9.03	8.01–10.04	100.00
**Panel B: Odds ratios by specialty (reference: Gynecology)**					
**Surgical Specialty**	**Odds Ratio**	**95% CI**	*P*-value		
Maxillofacial	4.78	2.39–9.58	< 0.001		
ENT	3.91	1.94–7.86	< 0.001		
Neurosurgery	2.41	1.09–5.29	0.030		
Abdominal	2.26	1.06–4.83	0.035		
Urology	1.65	0.68–4.00	0.270		
Orthopedic	1.44	0.60–3.42	0.414		
*Gynecology*	*Reference*	*—*	*—*		

Prevalence estimates with exact binomial 95% confidence intervals. Specialties ranked by DDL prevalence (descending). Odds ratios from binary logistic regression with Gynecology as reference. Bold values indicate statistical significance (α = 0.05)

*DDL *Difficult direct laryngoscopy, *CI *Confidence interval, *OR *Odds ratio, *ENT *Ear, nose, and throat

### Prevalence of DDL by surgical specialty

DDL prevalence varies substantially across surgical specialties (Table 2). Maxillofacial surgery had the highest DDL rate (13.53%; 95% CI: 11.33–16.08%), followed by ENT (11.34%; 95% CI: 9.31–13.73%), neurosurgery (7.28%; 95% CI: 4.12–12.57%), abdominal (6.90%; 95% CI: 4.91–9.63%), urology (5.08%; 95% CI: 3.15–8.09%), orthopedic (4.50%; 95% CI: 2.65–7.54%), and gynecology (3.17%; 95% CI: 1.68–5.91%).

Using gynecology as the reference category (lowest DDL prevalence), maxillofacial surgery carried the highest odds of DDL (OR 4.78; 95% CI: 2.39–9.58), followed by ENT (OR 3.91; 95% CI: 1.94–7.86), neurosurgery (OR 2.41; 95% CI: 1.09–5.29), and abdominal surgery (OR 2.26; 95% CI: 1.06–4.83) (Table 2). Orthopedic (OR 1.44; 95% CI: 0.60–3.42) and urology (OR 1.65; 95% CI: 0.68–4.00.68.00) did not differ significantly from gynecology. These findings confirm that the elevated overall DDL prevalence of 9.03% in this cohort is driven predominantly by the high proportion of head-and-neck procedures, which together comprised 51.7% of all cases.

### Univariate predictors of DDL

Table 3 presents the univariate associations between each bedside airway test and DDL. Six of the seven tested predictors achieved statistical significance (*p* < 0.05). Mallampati III/IV had the highest crude odds ratio (OR 4.03; 95% CI: 2.95–5.51), followed by BMI ≥ 27.5 kg/m² (OR 2.93; 95% CI: 2.28–3.77) and neck mobility less than 80 degrees (OR 2.13; 95% CI: 1.66–2.73). Neck circumference ≥ 40 cm (OR 1.39; 95% CI: 1.08–1.78), inter-incisor gap ≤ 3 cm (OR 1.36; 95% CI: 1.04–1.79), and thyromental distance ≤ 6.5 cm (OR 1.94; 95% CI: 1.47–2.56) were also significant. ULBT Grade III was not significantly associated with DDL (OR 1.18; 95% CI: 0.92–1.51; *p* = 0.183).

**Table 3:** Univariate predictors of DDL

Predictor (High-Risk Definition)	DDL Prevalence in High-Risk Group	Crude OR	95% CI	*P*-value
Mallampati class III/IV	13.37%	4.03	2.95–5.51	< 0.001
BMI ≥ 27.5 kg/m²	15.13%	2.93	2.28–3.77	< 0.001
Neck mobility < 80°	12.86%	2.13	1.66–2.73	< 0.001
TMD ≤ 6.5 cm	11.04%	1.94	1.47–2.56	< 0.001
NC ≥ 40 cm	10.83%	1.39	1.08–1.78	0.010
IIG ≤ 3 cm	9.85%	1.36	1.04–1.79	0.027
*ULBT Grade III*	9.76%	1.18	0.92–1.51	*0.183*

Bold rows indicate statistical significance (α = 0.05). The ULBT did not reach significance

*DDL *Difficult direct laryngoscopy, *OR* Odds ratio, *CI C*onfidence interval, *BMI* Body mass index, *TMD* Thyromental distance, *NC *Neck circumference, *IIG *Inter-incisor gap, *ULBT *Upper Lip Bite Test

### Multivariable analysis

**Fig. 2:**
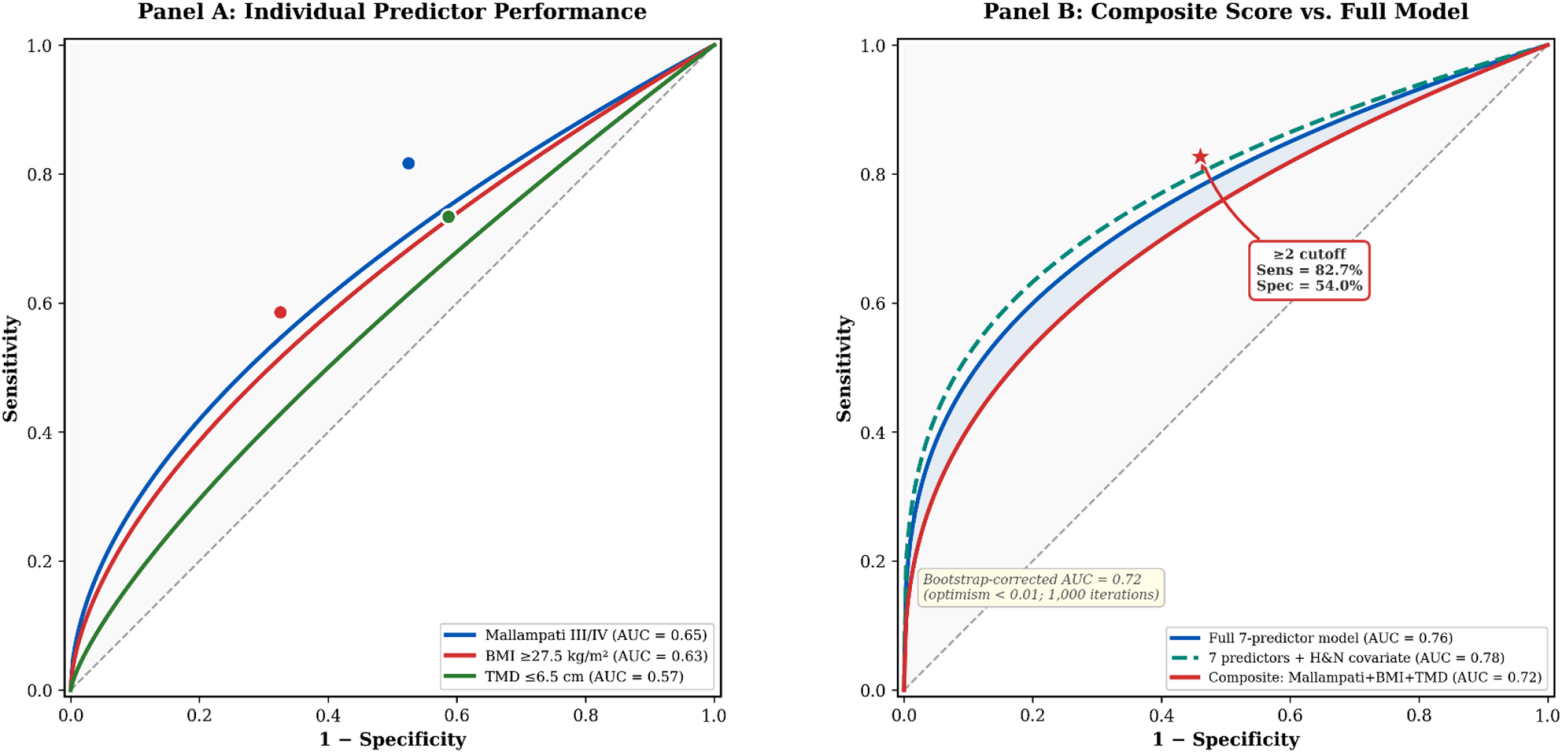
Receiver operating characteristic (ROC) analysis. Receiver operating characteristic (ROC) analysis for predicting difficult direct laryngoscopy. Panel **A** Individual predictor performance. Mallampati class III/IV achieved the highest individual AUC (0.65), followed by BMI ≥ 27.5 kg/m² (0.63) and TMD ≤ 6.5 cm (0.57). Solid circles indicate operating points at the high-risk cutoff. Panel **B** Composite score (Mallampati + BMI + TMD) versus the full 7-predictor model and the 8-predictor model adjusted for head-and-neck surgical category. The composite score (AUC 0.72) approaches the full model (AUC 0.76) with substantially fewer variables. The bootstrap-corrected estimate (AUC 0.72; optimism < 0.01) confirms minimal overfitting. The annotated star corresponds to the ≥ 2 cutoff (82.7% sensitivity, 54.0% specificity)

All seven predictors (including ULBT) were entered simultaneously into the multivariable logistic regression model. Model fit was acceptable (Hosmer–Lemeshow *p* = 0.42), and overall discrimination was fair (AUC 0.76; Fig. 2, Panel B). Multicollinearity was low (all VIF < 2). Six variables independently predicted DDL (Table 4): Mallampati III/IV (AOR 4.15; 95% CI: 3.02–5.70; *p* < 0.001), BMI ≥ 27.5 kg/m² (AOR 2.92; 95% CI: 2.25–3.79; *p* < 0.001), neck mobility less than 80 degrees (AOR 2.13; 95% CI: 1.64–2.75; *p* < 0.001), TMD ≤ 6.5 cm (AOR 1.95; 95% CI: 1.46–2.59; *p* < 0.001), NC ≥ 40 cm (AOR 1.41; 95% CI: 1.09–1.84; *p* = 0.010), and IIG ≤ 3 cm (AOR 1.41; 95% CI: 1.06–1.88; *p* = 0.018). ULBT Grade III remained non-significant in the adjusted model (AOR 1.02; 95% CI: 0.78–1.32; *p* = 0.912).

**Table 4:** Multivariable logistic regression: seven bedside predictors

Predictor	High-Risk Definition	AOR	95% CI	*P*-value
Mallampati score	Class III or IV	4.147	3.015–5.704	< 0.001
Body mass index	≥ 27.5 kg/m²	2.917	2.248–3.785	< 0.001
Neck mobility	< 80°	2.125	1.639–2.754	< 0.001
Thyromental distance	≤ 6.5 cm	1.948	1.463–2.592	< 0.001
Neck circumference	≥ 40 cm	1.412	1.086–1.836	0.010
Inter-incisor gap	≤ 3 cm	1.411	1.060–1.876	0.018
*Upper Lip Bite Test*	*Grade III*	*1.015*	*0.783–1.315*	*0.912*

All seven predictors entered simultaneously. Hosmer–Lemeshow *p* = 0.42; AUC = 0.76; all VIF < 2. Bold rows indicate independent statistical significance (α = 0.05)

*AOR* Adjusted odds ratio, *CI *Confidence interval

### Adjusted model with head-and-neck surgical covariate

To evaluate potential confounding by surgical case-mix, a second multivariable model included head-and-neck surgical category (ENT or maxillofacial versus all other specialties) as a binary covariate alongside the seven bedside predictors (Table 5). Head-and-neck surgery was independently associated with DDL (AOR 2.42; 95% CI: 1.83–3.21; *p* < 0.001). Critically, all six bedside predictors that were significant in the primary model retained independent significance after adjustment for surgical specialty, with minimal attenuation of effect sizes: Mallampati III/IV (AOR 4.07), BMI ≥ 27.5 kg/m² (AOR 2.91), neck mobility (AOR 2.13), TMD (AOR 1.94), NC (AOR 1.43), and IIG (AOR 1.39). ULBT remained non-significant (*p* = 0.990). The expanded model achieved an AUC of 0.78, and multicollinearity remained low (all VIF < 2). These results indicate that the bedside predictors capture airway-relevant anatomical features independent of the surgical indication itself.

**Table 5:** Multivariable logistic regression adjusted for head-and-neck surgical category.

Predictor	High-Risk Definition	AOR	95% CI	*P*-value
Mallampati score	Class III or IV	4.071	2.952–5.612	< 0.001
Body mass index	≥ 27.5 kg/m²	2.905	2.233–3.779	< 0.001
H&N surgery	ENT or Maxillofacial	2.423	1.829–3.209	< 0.001
Neck mobility	< 80°	2.132	1.641–2.771	< 0.001
Thyromental distance	≤ 6.5 cm	1.940	1.454–2.588	< 0.001
Neck circumference	≥ 40 cm	1.427	1.094–1.860	0.009
Inter-incisor gap	≤ 3 cm	1.387	1.040–1.850	0.026
*Upper Lip Bite Test*	*Grade III*	*0.998*	*0.768–1.297*	*0.990*

Model includes seven bedside predictors plus H&N surgical category as a binary covariate. AUC = 0.78; all VIF < 2. All six bedside predictors retain significance after adjustment for surgical specialty

*AOR *Adjusted odds ratio, *CI *Confidence interval, *H&N *Head-and-neck

### Performance of the composite score

The simplified composite score (summing Mallampati, BMI, and TMD; range 0–3) demonstrated superior discrimination compared any single predictor (Table 6). The composite score achieved an AUC of 0.72 (95% CI: 0.69–0.75; bootstrap-corrected 0.72, optimism < 0.01). The score exhibited a clear dose–response relationship: DDL prevalence increased progressively from 2.2% at score 0 to 3.3% at score 1, 11.9% at score 2, and 24.7% at score 3. At a cutoff of 2 or greater, the score yielded a sensitivity of 82.7% (95% CI: 77.9–86.7%), specificity of 54.0% (95% CI: 52.1–55.8%), positive predictive value of 15.1% (95% CI: 13.4–17.0%), and negative predictive value of 96.9% (95% CI: 95.9–97.7%), with a positive likelihood ratio of 1.80 and a negative likelihood ratio of 0.32 (Table 7). The high NPV indicates that a score of 0 or 1 provides substantial reassurance against DDL. At the more stringent cutoff of 3 (all three risk factors present), specificity rose to 89.8% but sensitivity fell to 33.8%, identifying a smaller but higher-risk subgroup (24.7% DDL prevalence).

**Table 6:** Composite score performance: individual versus combined models.

Model	AUC	Sensitivity, %	Specificity, %	95% CI for AUC
Mallampati + BMI + TMD (≥ 2)	0.72	82.7	54.0	0.69–0.75
Mallampati alone	0.65	81.7*	47.5*	—
BMI alone	0.63	58.6*	67.4*	—
TMD alone	0.57	73.4*	41.3*	—

*AUC *Area under the ROC curve, *BMI* Body mass index, *TMD* Thyromental distance

*Operating point at the binary high-risk cutoff for individual predictors. The combined model (bold) demonstrated the highest discriminative performance. Internal validation via bootstrap resampling (1,000 iterations) yielded a corrected AUC of 0.72 (optimism < 0.01)

**Table 7:** Diagnostic accuracy of the composite score (≥ 2 cutoff) for predicting DDL (*N* = 3,080)

Metric	Value	95% CI	Clinical Interpretation
True positives (TP)	230	—	DDL correctly identified as high risk
False positives (FP)	1,290	—	Non-DDL incorrectly flagged
True negatives (TN)	1,512	—	Non-DDL correctly classified as low risk
False negatives (FN)	48	—	DDL missed by score (17.3% of DDL cases)
Sensitivity	82.7%	77.9–86.7%	Over 4 in 5 DDL cases detected
Specificity	54.0%	52.1–55.8%	Moderate; trades specificity for sensitivity
PPV	15.1%	13.4–17.0%	~ 1 in 7 screen-positive patients has DDL
NPV	96.9%	95.9–97.7%	Score < 2 rules out DDL in > 96% of cases
LR+	1.80	—	Modest increase in post-test probability
LR−	0.32	—	Meaningful decrease; useful for rule-out

The composite score assigns 1 point each for Mallampati class III/IV, BMI ≥ 27.5 kg/m², and TMD ≤ 6.5 cm (range 0–3). CIs calculated using the Wilson score method. Predictive values are prevalence-dependent

*DDL* Difficult direct laryngoscopy, *CI* Confidence interval, *LR* Likelihood ratio, *PPV *Positive predictive value, *NPV *Negative predictive value

### Sensitivity analyses

#### Restriction to non-head-and-neck cases

After excluding ENT and maxillofacial cases, the non-head-and-neck subgroup comprised 1,488 patients with 80 DDL events (5.38%; 95% CI: 4.30–6.64%), confirming that the elevated overall prevalence is substantially driven by the center’s specialty case-mix. In this subgroup, the composite score retained fair discrimination (AUC 0.75). The three composite-score predictors (Mallampati III/IV, BMI ≥ 27.5 kg/m², TMD ≤ 6.5 cm) and neck mobility remained independently significant (all *p* < 0.05), whereas neck circumference (*p* = 0.29) and inter-incisor gap (*p* = 0.046) showed attenuated significance. Notably, BMI emerged as the strongest predictor in the non-head-and-neck subgroup (AOR 4.18), exceeding Mallampati (AOR 3.36), likely reflecting the reduced contribution of tumor-related anatomical distortion in this surgical population.

#### Restriction to Cormack–Lehane III/IV definition

When DDL was defined solely by Cormack–Lehane grade III or IV (excluding 25 cases classified as DDL based only on three or more attempts), 253 patients (8.21%) met this stricter criterion. The composite score AUC was 0.72, and all six original independent predictors retained statistical significance with comparable effect sizes (e.g., Mallampati AOR 3.99). IIG lost significance under this definition (*p* = 0.052), and the ULBT remained non-significant (*p* = 0.615).

#### Specialty-specific composite score performance

The composite score demonstrated consistent discrimination across surgical subgroups (Table 8). AUC values ranged from 0.68 in gynecology to 0.82 in orthopedic surgery, with the score performing comparably in head-and-neck cases (AUC 0.71; *n* = 1,592; DDL = 198) and non-head-and-neck cases (AUC 0.75; *n* = 1,488; DDL = 80). These results support the score’s applicability beyond the specialty-heavy case-mix of this institution, although subgroup estimates should be interpreted with caution given the limited number of DDL events in some specialties.

**Table 8:** Composite score AUC by surgical specialty subgroup

Subgroup	*n*	DDL events	AUC	Interpretation
Orthopedic	289	13	0.82	Highest; limited events
Urology	315	16	0.80	Good; limited events
Non-H&N aggregate	1,488	80	0.75	Fair; adequate events
Neurosurgery	151	11	0.75	Fair; limited events
ENT	794	90	0.72	Fair; adequate events
H&N aggregate	1,592	198	0.71	Fair; adequate events
Abdominal	449	31	0.71	Fair; moderate events
Maxillofacial	798	108	0.70	Fair; adequate events
Gynecology	284	9	0.68	Lower; Fewest events
Overall	3,080	278	0.72	

Subgroups ranked by AUC in descending order. Subgroups with fewer than 20 DDL events should be interpreted with caution due to limited statistical power

*AUC* Area under the ROC curve, *H&N *Head-and-neck, *DDL* Difficult direct laryngoscopy

## Discussion

### Overview of key findings

This retrospective cohort study of 3,080 adult elective surgical patients at a Cambodian tertiary referral center demonstrated a DDL prevalence of 9.03% (95% CI: 8.01–10.04%) and identified six independent bedside predictors. A simplified three-component composite score (Mallampati class + BMI + TMD) showed fair discrimination (AUC 0.72; 95% CI: 0.69–0.75) that was superior to any individual predictor, offering a practical screening tool for resource-limited settings. When head-and-neck surgical category was added as a covariate, all six bedside predictors retained independent significance, confirming that the score captures airway-relevant anatomical features rather than merely reflecting surgical case-mix.

### Prevalence of DDL

The observed 9.03% prevalence lies within the broad global range (1.5–18%) but notably exceeds rates reported in several high-resource European and North American datasets, such as the DIFFICAIR cohort in Denmark (1.87%) and a German multicenter study of 102,305 cases (4.9%) [8, 33]. It is comparable to rates in other low- and middle-income settings, including Ethiopia (12.2%) [9], and exceeds the Thai estimate of 3.2% [34]. A systematic review and meta-analysis by Wang et al. reported a pooled global prevalence of 5.51% [35]. The difference between our cohort and many published series is primarily attributable to the distinctive case-mix at Preah Ang Duong Hospital, where 51.7% of cases were ENT or maxillofacial procedures — specialties intrinsically associated with higher DDL risk due to tumors, goiters, and facial trauma causing anatomical distortion [5, 22–24, 36–39]. The sensitivity analysis restricting the cohort to non-head-and-neck cases yielded a prevalence of 5.38%, much closer to the global pooled estimate, supporting this interpretation.

### Independent predictors of DDL

The six independent predictors identified in this study are broadly consistent with prior literature, though the relative contribution of BMI is notably stronger in our cohort than in several published series.

Mallampati class III/IV was the strongest predictor (AOR 4.15; 95% CI: 3.02–5.70), corroborating extensive evidence from meta-analyses and international cohorts [16, 40, 41]. However, Mallampati alone has well-documented sensitivity limitations (81.7% in our data at the binary cutoff, but with only 47.5% specificity), reinforcing the need for composite assessment rather than reliance on any single test. The Shiga meta-analysis reported a pooled sensitivity of 49% and specificity of 86% for Mallampati as a standalone predictor [16], further supporting its use as one component within a multifactor score.

BMI ≥ 27.5 kg/m² emerged as the second strongest independent predictor (AOR 2.92; 95% CI: 2.25–3.79), a stronger association than reported in several prior studies. For comparison, Juvin et al. reported an odds ratio of 1.6 for difficult intubation in obese versus lean patients [40], and the German multicenter study found BMI to be a modest predictor (OR 1.3–1.5) [33]. The stronger effect in our cohort likely reflects the use of the WHO Asian-specific obesity threshold (27.5 kg/m²), which captures a physiologically relevant level of adipose tissue deposition that may be missed by the standard threshold of 30 kg/m² used in most published studies. At this lower cutoff, 35.0% of our cohort was classified as obese, and DDL prevalence in this group was 15.1% versus 5.8% in non-obese patients. The finding that BMI became the strongest predictor in the non-head-and-neck subgroup (AOR 4.18) further underscores its importance in populations where tumor-related anatomical distortion is less prevalent. These results support the use of population-specific BMI thresholds in airway risk stratification.

Restricted neck mobility emerged as the third strongest predictor (AOR 2.13; 95% CI: 1.64–2.75), consistent with its physiological role in aligning the oral, pharyngeal, and laryngeal axes required for direct visualization of the glottis [10]. TMD ≤ 6.5 cm (AOR 1.95; 95% CI: 1.46–2.59) reflects restricted mandibular space that limits blade displacement during laryngoscopy [42, 43]. NC ≥ 40 cm (AOR 1.41; 95% CI: 1.09–1.84) captures the effects of adipose and soft-tissue deposition on upper airway landmarks and compressibility, consistent with findings from both obese and non-obese cohorts [33, 37]. IIG ≤ 3 cm (AOR 1.41; 95% CI: 1.06–1.88) reflects limited mouth opening that restricts laryngoscope blade insertion.

The non-significance of the Upper Lip Bite Test (*p* = 0.912 in the adjusted model) deserves particular attention. ULBT has shown variable performance across populations and study designs. Several factors may explain this finding in our cohort. First, ULBT performance depends heavily on patient cooperation and dental status; populations with high rates of dental pathology or edentulism may yield unreliable ULBT grades, and dental status was not systematically recorded in our dataset. Second, the test may have limited discriminatory power in cohorts where other dominant predictors — particularly Mallampati and neck mobility — capture overlapping anatomical information about oropharyngeal and submandibular space. Third, inter-observer variability in ULBT grading may be amplified in a retrospective setting where grading was not standardized across assessors. This finding is consistent with reports by Selvi et al. [44] and others questioning ULBT’s added value in multivariate models, and with the Cochrane review by Roth et al. [45–47] which found inconsistent performance across bedside airway tests.

### Effect of surgical specialty on predictor validity

A key concern raised during review was whether the identified bedside predictors merely reflect the higher baseline DDL risk inherent to head-and-neck surgery rather than capturing independent anatomical risk. To address this, we performed an additional multivariable model including H&N surgical category as a binary covariate. H&N surgery was independently associated with DDL (AOR 2.42; 95% CI: 1.83–3.21; *p* < 0.001), confirming that surgical specialty contributes to DDL risk beyond what the bedside predictors capture — likely through mechanisms such as tumor-induced tracheal deviation, submucosal edema, and radiation fibrosis that are not directly measured by the seven assessed predictors. Critically, all six previously significant bedside predictors retained independent significance with minimal attenuation of effect sizes (e.g., Mallampati AOR decreased from 4.15 to 4.07; BMI from 2.92 to 2.91). This demonstrates that the composite score identifies airway-relevant anatomical features that predict DDL regardless of surgical context, supporting its applicability across surgical populations [48].

### Composite score performance and clinical interpretation

The composite score (Mallampati + BMI + TMD) achieved an AUC of 0.72 (95% CI: 0.69–0.75; bootstrap-corrected 0.72, optimism < 0.01), representing fair discrimination. At the optimal cutoff of ≥ 2, the score achieved 82.7% sensitivity and 54.0% specificity, with a negative predictive value of 96.9%. The score exhibited a clear dose–response relationship: DDL prevalence rose from 2.2% at score 0 to 24.7% at score 3.

It is important to interpret this performance honestly. At the ≥ 2 cutoff, approximately 48 of 278 DDL cases (17.3%) would be classified as low risk and would not trigger enhanced airway preparation. While this represents an improvement over single-predictor screening (Mallampati alone misses approximately 18.3% of DDL cases at its binary cutoff), any missed difficult airway carries potential for serious harm. The score should therefore be understood as a screening and stratification tool, not as a stand-alone diagnostic test. Its primary value lies in identifying a higher-risk subgroup warranting enhanced preparation (video laryngoscope availability, senior anesthesiologist presence, pre-induction team briefing), while routine vigilance and standard difficult-airway protocols should remain in place for all patients regardless of score. The high NPV (96.9%) means that patients scoring 0 or 1 can be managed with standard preparation with substantial — but not absolute — reassurance.

The equal-weighting approach (one point per predictor) was chosen for simplicity, given that the primary target users are anesthesia providers in resource-limited settings where complex weighted algorithms may reduce clinical uptake. However, the wide range of AORs across the three components (1.95 to 4.15) suggests that a weighted score — for example, assigning 2 points for Mallampati and 1 point each for BMI and TMD — might improve discrimination modestly and should be explored in future validation studies.

### Comparison with existing composite scores

Several multifactor airway prediction tools have been proposed in the literature, and it is important to contextualize our score relative to these. The Simplified Airway Risk Index (SARI) incorporates seven factors (mouth opening, thyromental distance, Mallampati class, neck mobility, jaw protrusion ability, body weight, and history of difficult intubation) and has reported AUCs ranging from 0.72 to 0.78 in validation studies [31]. The LEMON assessment (Look, Evaluate 3-3-2 rule, Mallampati, Obstruction, Neck mobility) is widely taught in emergency medicine but was designed for clinical decision-making rather than quantitative scoring and has limited validation data [30]. The Wilson risk sum uses five factors with weighted scoring and achieved an AUC of approximately 0.75 in its derivation cohort [29, 49]. The El-Ganzouri multivariate risk index incorporates seven variables with weighted points and reported sensitivity of 66% and specificity of 84% [50].

Our Mallampati + BMI + TMD score (AUC 0.72) performs comparably to these established tools despite using substantially fewer components (three versus five to seven) and requiring no specialized equipment. The SARI and El-Ganzouri scores, while slightly more discriminative, require assessment of jaw protrusion ability and prior intubation history — information that may not be available in first-presentation elective patients or in settings where medical records are incomplete. The principal differentiating factor of our score is its zero-cost feasibility: the three components require only a ruler, a scale, and a visual oropharyngeal assessment, making it implementable in any clinical environment regardless of resource availability. Whether this practical advantage justifies the modest reduction in discrimination compared with more complex tools is ultimately a question of clinical context and should be evaluated in prospective implementation studies.

### Differentiating factors of the Cambodian cohort

Several features distinguish this cohort from most published airway prediction studies and may explain observed differences in predictor performance. First, the heavy representation of head-and-neck surgery (51.7%) is unusual; most published cohorts are drawn from general surgical or mixed populations where H&N cases comprise 5–15% of the case-mix. This inflates the baseline DDL prevalence and may amplify the apparent effect of predictors that correlate with surgical pathology (e.g., NC, Mallampati). The H&N-adjusted model addresses this concern directly. Second, the use of the WHO Asian-specific BMI threshold (27.5 kg/m²) rather than the standard 30 kg/m² cutoff enhances the population relevance of the BMI component but limits direct comparison with studies using higher thresholds. Third, the reliance on clinical estimation for neck mobility (rather than goniometric measurement) reflects real-world practice in resource-limited settings but introduces measurement variability that may attenuate the observed association. Fourth, the Cambodian population’s craniofacial morphology, body habitus, and disease profile may differ meaningfully from those in European, North American, and even other Southeast Asian populations, underscoring the need for population-specific validation of any airway prediction tool.

### Demographic findings

Our cohort demographics (mean age 53.2 years, near-equal sex distribution, mean BMI 26.5 kg/m² with 35.0% classified as obese by Asian cutoffs) broadly align with regional reports. Neither age (*p* = 0.350) nor sex (*p* = 0.580) differed significantly between DDL and non-DDL groups, and neither was an independent predictor in our models, consistent with some but not all prior studies. ASA physical status showed a striking dose–response relationship with DDL (4.9% in ASA I versus 23.5% in ASA IV; *p* < 0.001), consistent with higher comorbidity burden conferring greater airway management complexity. ASA class was not included in the composite score because it reflects systemic illness severity rather than airway-specific anatomy, but it serves as a useful clinical correlate for overall risk stratification.

### Strengths

This study has several notable strengths. It represents the largest airway assessment study conducted in Cambodia and one of the few from Southeast Asia, addressing a genuine knowledge gap. The sample size provides approximately 40 events per variable, exceeding conventional thresholds for stable logistic regression. The use of population-specific BMI cutoffs enhances relevance to the target population. The study evaluates multiple bedside predictors simultaneously and proposes a zero-cost screening tool suitable for resource-limited settings. The inclusion of a head-and-neck surgical covariate model, specialty-specific AUC analysis, bootstrap internal validation, and two additional sensitivity analyses provides a thorough assessment of model robustness. The inclusion of a detailed patient flow diagram, STROBE checklist, and clear outcome definitions enhances transparency and reproducibility.

### Limitations

Several limitations merit consideration and should temper interpretation of our findings. First, the retrospective design depended on routine clinical documentation, introducing risks of measurement variability and documentation bias. Cormack–Lehane grading was performed by multiple anesthesiologists without standardized calibration, and inter-rater reliability was not assessed; this may have led to misclassification of the outcome in some cases. The use of clinical visual estimation for neck mobility, rather than goniometric measurement, introduces additional inter-observer variability. Second, this is a single-center study with a case-mix heavily weighted toward head-and-neck surgery (51.7%), substantially limiting generalizability to general surgical populations or other hospital types. However, the sensitivity analysis in non-H&N cases (AUC 0.75) and the H&N-adjusted model provide partial reassurance [51, 52]. Third, 224 patients (7.3%) were excluded for missing data; residual selection bias cannot be excluded. Fourth, the composite score was derived and evaluated on the same dataset without external validation. The bootstrap-corrected AUC (0.72, optimism < 0.01) provides some internal validation, but prospective testing in independent cohorts is essential before clinical implementation. Fifth, residual confounding may persist despite adjustment; variables such as specific pathological diagnosis (cancer type, fracture classification), tumor size, airway compression, prior radiation therapy, operator experience level, and comorbidity burden were not available in the dataset. Sixth, the composite score uses equal weighting despite substantially different AORs among its components, which may be suboptimal. Seventh, the study did not assess downstream clinical outcomes (e.g., failed intubation, emergency surgical airway, aspiration events), so the relationship between DDL prediction and patient outcomes cannot be evaluated. Eighth, ASA class and surgical indication data were available but could not be included as covariates in the composite score derivation; their potential contribution to prediction should be explored in future studies.

### Clinical implications

Despite its limitations, the composite score has practical appeal for its target setting. The three components (Mallampati, BMI, TMD) require no specialized equipment and can be performed in under two minutes during routine preoperative assessment. In settings where video laryngoscopes and fiberoptic equipment are not readily available, identifying high-risk patients preoperatively allows anesthesia teams to assemble rescue equipment, request senior support, consider awake intubation techniques, and mentally rehearse contingency plans [53, 54]. A score of 2 or greater could be embedded in pre-anesthesia checklists to trigger a standardized escalation pathway. However, it must be emphasized that no bedside score can replace a comprehensive airway assessment strategy, and even patients scoring 0 or 1 may present unexpected difficulty — standard difficult-airway equipment should remain accessible for all cases.

## Conclusion

This retrospective cohort study from Preah Ang Duong Hospital quantified the burden of difficult direct laryngoscopy among 3,080 adult elective surgical patients and identified six independent bedside predictors. DDL occurred in 9.03% of patients (95% CI: 8.01–10.04%), a prevalence driven by the center’s high volume of head-and-neck procedures; maxillofacial (13.53%) and ENT (11.34%) surgeries had the highest rates. Six bedside factors were independently associated with DDL: Mallampati III/IV (AOR 4.15; 95% CI: 3.02–5.70), BMI ≥ 27.5 kg/m² (AOR 2.92; 95% CI: 2.25–3.79), limited neck mobility (AOR 2.13; 95% CI: 1.64–2.75), TMD ≤ 6.5 cm (AOR 1.95; 95% CI: 1.46–2.59), NC ≥ 40 cm (AOR 1.41; 95% CI: 1.09–1.84), and IIG ≤ 3 cm (AOR 1.41; 95% CI: 1.06–1.88). The Upper Lip Bite Test was not independently predictive (*p* = 0.91). All six predictors retained significance after adjustment for head-and-neck surgical category, confirming that the score captures airway-specific anatomical risk independent of surgical case-mix.

A simplified, equally weighted three-component composite score (Mallampati + BMI + TMD) demonstrated fair discrimination (AUC 0.72; 95% CI: 0.69–0.75; bootstrap-corrected 0.72) with 82.7% sensitivity, 54.0% specificity, 96.9% negative predictive value, and a clear dose–response relationship at a cutoff of 2 or greater, outperforming any individual predictor. The score performed consistently across surgical subgroups (specialty-specific AUCs 0.68–0.82), including in non-head-and-neck cases (AUC 0.75). Age and sex were not independent predictors. BMI emerged as a notably strong predictor (second only to Mallampati) when assessed using the WHO Asian-specific obesity threshold, supporting the use of population-appropriate cutoffs in airway risk stratification.

However, the composite score was derived and evaluated on the same dataset without external validation, and the single-center, specialty-heavy case-mix limits generalizability. The modest specificity at the ≥ 2 cutoff (54.0%) means that nearly half of screen-positive patients will not have DDL, and approximately 17% of DDL cases will be missed. These performance characteristics characterize the score as a screening and stratification aid rather than a definitive diagnostic tool; standard difficult-airway equipment and protocols should remain available for all patients regardless of score. Prospective, multicenter validation studies—ideally including diverse surgical populations across Southeast Asian institutions—are essential before this score can be recommended for routine clinical implementation. Future research should also explore weighted scoring approaches, evaluate whether the score’s use translates into improved patient outcomes, and investigate objective adjuncts such as point-of-care ultrasound-based airway evaluation to further refine prediction.

## Supplementary Information

Supplementary Material 1: Table S1. STROBE Statement — Checklist of items that should be included in reports of cohort studies.

## Data Availability

De-identified dataset, analysis code, and the data dictionary are available from the corresponding author on reasonable request, in accordance with institutional policy and ethics approval.
