# Supplementary material for: Prevalence and bedside predictors of difficult direct laryngoscopy among 3,080 adult elective surgical patients at a Cambodian tertiary center: a retrospective cohort study
Source: BMC Anesthesiol. 2026 Apr 24;26:337. doi: 10.1186/s12871-026-03846-4 (PMC13221751; doi:10.1186/s12871-026-03846-4)
Supplement: Supplementary file 1 — Supplementary Material 1: Table S1. STROBE Statement — Checklist of items that should be included in reports of cohort studies. [file 12871_2026_3846_MOESM1_ESM.docx]

## **Table S1. STROBE Statement** — Checklist of items that should be included in reports of cohort studies.

*von Elm E, Altman DG, Egger M, Pocock SJ, Gøtzsche PC, Vandenbroucke JP; STROBE Initiative. Ann Intern Med. 2007;147(8):573-577.*

| **Item No.** | **STROBE Item** | **Page** | **Comment / Location in Manuscript** |
| --- | --- | --- | --- |
| **Title and Abstract** |  |  |  |
| 1 | Title/Abstract: (a) Indicate the study’s design with a commonly used term in the title or abstract; (b) Provide an informative and balanced summary | 1–3 | Study design stated in title (“retrospective cohort study”). Abstract provides structured summary with Background, Methods, Results, Conclusions. |
| **Introduction** |  |  |  |
| 2 | Background/rationale: Explain the scientific background and rationale for the investigation being reported | 4–5 | Introduction paragraphs 1–4: DDL significance, prevalence variability, bedside tool limitations, Southeast Asian literature gap. |
| 3 | Objectives: State specific objectives, including any prespecified hypotheses | 5 | Three objectives stated: (i) prevalence, (ii) independent predictors, (iii) composite score discrimination. “Objectives” paragraph. |
| **Methods** |  |  |  |
| 4 | Study design: Present key elements of study design early in the paper | 6 | Retrospective cohort study, January–June 2023. “Study Design and Setting” subsection. |
| 5 | Setting: Describe the setting, locations, and relevant dates, including periods of recruitment, exposure, follow-up, and data collection | 6 | Preah Ang Duong Hospital, Phnom Penh, Cambodia. Study period: 1 January–30 June 2023. |
| 6 | Participants: (a) Give eligibility criteria, sources and methods of selection; (b) For cohort studies, describe follow-up | 6 | (a) Adults ≥18 years, elective surgery, planned Macintosh DL. Exclusion criteria detailed. (b) Single cross-sectional assessment; no longitudinal follow-up. Figure 1 (flowchart). |
| 7 | Variables: Clearly define all outcomes, exposures, predictors, potential confounders, and effect modifiers | 7–8 | DDL outcome defined (C-L III/IV or ≥3 attempts). Seven bedside predictors defined with measurement techniques, instruments, anatomical landmarks, and high-risk cutoffs. H&N surgical category defined as covariate. ASA and indication variables specified. |
| 8 | Data sources/measurement: For each variable, give sources of data and details of methods of assessment (measurement). Describe comparability of assessment methods if there is more than one group | 7–8 | Standardized departmental anesthesia record described. Measurement technique for each predictor specified (Mallampati: seated, no phonation; TMD: rigid ruler, mouth closed, head extended; NC: flexible tape at thyroid cartilage; IIG: ruler/caliper; neck mobility: clinical visual estimation; ULBT: I–III grading). Inter-rater reliability limitation acknowledged. |
| 9 | Bias: Describe any efforts to address potential sources of bias | 6, 28–29 | Excluded patient comparison attempted (age, sex). Trainee supervision and attending C-L confirmation described. Limitations section addresses documentation bias, measurement variability, selection bias from missing data, single-center design, and residual confounding. |
| 10 | Study size: Explain how the study size was arrived at | 11 | Sample size considerations: 278 DDL events / 7 predictors = EPV ≈40, exceeding minimum 10–20 EPV threshold [51]. |
| 11 | Quantitative variables: Explain how quantitative variables were handled in the analyses. If applicable, describe which groupings were chosen and why | 7–8 | All predictors dichotomized at a priori clinically established cutoffs (e.g., BMI ≥27.5 kg/m² per WHO Asian threshold; TMD ≤6.5 cm; NC ≥40 cm). Rationale provided for each cutoff. |
| 12 | Statistical methods: (a) Describe all statistical methods; (b) Describe any methods used to examine subgroups and interactions; (c) Explain how missing data were addressed; (d) Describe any sensitivity analyses | 9–11 | (a) Chi-square/Fisher’s exact for univariate; multivariable logistic regression; ROC analysis; bootstrap validation (1,000 iterations); Hosmer–Lemeshow calibration. Logistic regression justified over alternatives. SPSS V30 specified. (b) Specialty-specific AUC subgroup analyses (Table 8). H&N-adjusted model (Table 5). (c) 224 patients excluded for missing data; demographic comparison performed. (d) Three pre-specified sensitivity analyses: non-H&N subgroup, C-L III/IV–only definition, specialty-specific AUCs. |
| **Results** |  |  |  |
| 13 | Participants: (a) Report numbers of individuals at each stage of study; (b) Give reasons for non-participation at each stage; (c) Consider use of a flow diagram | 11–13, Fig. 1 | (a–b) Numbers at each stage with exclusion reasons: 12,221 → 6,760 → 5,646 → 3,080. (c) Figure 1 (study flowchart) provided. |
| 14 | Descriptive data: (a) Give characteristics of study participants; (b) Indicate number of participants with missing data for each variable of interest | 11–12, Table 1 | (a) New Table 1: age, sex, BMI, ASA class, specialty, all 7 predictors, stratified by DDL status with p-values. (b) 224 excluded for missing data (7.3%); all included patients have complete predictor data. |
| 15 | Outcome data: Report numbers of outcome events or summary measures | 13 | DDL: 278/3,080 (9.03%; 95% CI: 8.01–10.04%). Breakdown: 253 by C-L III/IV, 25 by ≥3 attempts only. Specialty-specific prevalence in Table 2. |
| 16 | Main results: (a) Give unadjusted estimates and, if applicable, confounder-adjusted estimates and their precision; (b) Report category boundaries when continuous variables were categorized; (c) If relevant, consider translating estimates into meaningful clinical terms | 14–18 | (a) Univariate ORs (Table 3) and adjusted ORs (Table 4) with 95% CIs and p-values. H&N-adjusted model (Table 5). (b) All cutoffs specified (e.g., BMI ≥27.5, TMD ≤6.5 cm). (c) Composite score dose–response (2.2% → 24.7% DDL across score 0–3); PPV/NPV with clinical interpretation (Table 7). |
| 17 | Other analyses: Report other analyses done—e.g., analyses of subgroups and interactions, and sensitivity analyses | 19–20 | Three sensitivity analyses (Table 8): (1) non-H&N subgroup (n=1,488; AUC 0.75); (2) C-L III/IV–only definition (AUC 0.72); (3) specialty-specific AUCs (range 0.68–0.82). Bootstrap internal validation (AUC 0.72, optimism <0.01). |
| **Discussion** |  |  |  |
| 18 | Key results: Summarise key results with reference to study objectives | 21 | Opening paragraph summarizes prevalence (9.03%), six independent predictors, composite score AUC (0.72), and H&N adjustment findings. |
| 19 | Limitations: Discuss limitations of the study, taking into account sources of potential bias or imprecision | 28–29 | Eight limitations discussed: retrospective design, inter-rater variability (including clinical estimation for neck mobility), single-center specialty-heavy case-mix, missing data, lack of external validation, residual confounding (specific diagnosis, tumor size, radiation, operator experience), equal weighting, and no downstream outcome assessment. |
| 20 | Interpretation: Give a cautious overall interpretation of results considering objectives, limitations, multiplicity of analyses, results from similar studies, and other relevant evidence | 21–29 | Prevalence contextualized against global data. Predictors compared with meta-analyses and international cohorts. Composite score compared with SARI, LEMON, Wilson, El-Ganzouri. Cambodia-specific differentiating factors discussed. False-negative rate (17.3%) explicitly quantified with cautionary note. Score positioned as screening aid, not diagnostic tool. |
| 21 | Generalisability: Discuss the generalisability (external validity) of the study results | 21–29 | Generalizability limited by single-center, specialty-heavy case-mix (51.7% H&N). Non-H&N sensitivity analysis (AUC 0.75) provides partial reassurance. H&N-adjusted model confirms predictor independence from surgical context. Population-specific factors (Asian BMI threshold, craniofacial morphology) discussed. Multicenter validation recommended. |
| **Other Information** |  |  |  |
| 22 | Funding: Give the source of funding and the role of the funders for the present study and, if applicable, for the original study on which the present article is based | 31 | No specific funding received. Declarations section. |
